# Personalized AI for workplace health promotion: performance management and healthcare worker engagement through digital analytics

**DOI:** 10.3389/fpubh.2025.1718474

**Published:** 2026-01-13

**Authors:** Daniele Virgillito, Caterina Ledda

**Affiliations:** 1 Department of Economics and Business, University of Catania, Catania, Italy; 2 Department of Clinical and Experimental Medicine, University of Catania, Catania, Italy

**Keywords:** artificial intelligence, healthcare workers, occupational medicine, organizational efficiency, performance management, preventive medicine, workplace health promotion

## Abstract

**Background:**

Artificial intelligence (AI) is increasingly being applied in healthcare work-places to promote worker wellbeing and optimize organizational performance. However, evidence on its effectiveness, adoption, and limitations remains fragmented. This scoping review aimed to systematically map the literature on AI-based digital technologies for workplace health promotion and performance management among healthcare workers.

**Methods:**

The review was reported in accordance with PRISMA-ScR guidelines and was conducted up to July 2025. Studies were screened and selected using the PCC (Population–Concept–Context) framework, and data were extracted on AI technology type, health promotion focus, and outcomes. Electronic searches were conducted in PubMed, Scopus, Web of Science, PsycINFO, IEEE Xplore, and Google Scholar. The search identified 351 records; after removing duplicates and non-eligible papers, 180 records were screened, 84 full texts assessed, and 21 studies included in the final synthesis.

**Results:**

Twenty-one studies were included, covering quantitative, qualitative, and mixed-method designs. Two major domains of application emerged: AI-enabled health monitoring and intervention and AI-driven performance optimization. Reported benefits included reductions in stress, burnout, anxiety, and musculoskeletal pain, as well as improvements in workflow efficiency, documentation quality, leadership support, and staff engagement. However, limitations included short study durations, methodological heterogeneity, privacy and ethical concerns, and variable adoption by healthcare staff.

**Conclusions:**

AI-based digital technologies show promise for enhancing both worker health and organizational sustainability. To ensure long-term impact, future research should prioritize rigorous study designs, standardized outcome measures, privacy-preserving frameworks, and human-centered approaches to technology integration.

## 1 Introduction

Artificial intelligence (AI) and data analytics are reshaping workplace wellness and performance management across sectors. In human resource management, AI enhances engagement, productivity, and talent management through personalization and predictive analytics (1). In healthcare, digitalization and AI support efficiency and wellbeing of healthcare staff (2), while analytics-driven systems optimize engagement, retention, and performance conversations (3, 4). AI is also being applied to workplace mental health, offering monitoring, real-time feedback, and unbiased assessments (5, 6). Yet challenges such as privacy, ethical implications, and psychological effects underline the need for balanced integration of technology and human oversight (7).

The use of wearable devices, mobile health (mHealth) applications, and AI-driven analytics is particularly relevant in healthcare. These tools enable real-time monitoring, predictive insights, and personalized interventions (8, 9), contributing to early detection, proactive health management, and better outcomes (10). When applied to staff settings, such tools help monitor occupational stress, burnout, or work performance. Evidence shows their potential in promoting physical activity and weight control (11), and integration with human capital systems provides a broader view of workforce health (12). Nonetheless, privacy, limited personalization, and interoperability remain critical barriers (13).

Emerging studies suggest that AI-based tools can support workplace health promotion and performance management initiatives directed at healthcare workers. Chatbots, for example, improve adherence and engagement in self-monitoring (14, 15), though evidence on injury and illness prevention is limited (16). Broader health promotion programs in healthcare have reported short-term benefits in staff wellbeing, work ability, and reduced burnout (17, 18), with AI offering opportunities for greater personalization and risk prediction (19). However, the long-term effectiveness and methodological robustness of such interventions remain to be established (20).

Despite clear potential, the implementation of AI-based digital solutions is shaped by barriers and facilitators. Privacy and data security, lack of transparency, and ethical concerns persist (21–23), while personalization, efficiency, and improved accuracy act as enablers (24). Trust, education, and governance structures emerge as decisive for adoption (21, 25). The COVID-19 pandemic has further accelerated digital health adoption, highlighting the need for human-centered design and careful attention to organizational dynamics (26).

Given the complexity of AI adoption in healthcare settings, this review adopts a multidisciplinary perspective that integrates insights from occupational health, digital health, organizational behavior, and health informatics. This approach allows for a more comprehensive understanding of how AI technologies are implemented, experienced, and evaluated in relation to the healthcare workforce.

In this context, the objective of this scoping review is to systematically map the existing evidence on AI-based digital technologies implemented to support both performance management and workplace health promotion among healthcare workers. These two domains are increasingly interconnected, as AI-enabled systems often aim to improve individual wellbeing, such as reducing stress, anxiety, and burnout, while simultaneously enhancing organizational outcomes like efficiency, workflow optimization, and staff performance. The review examines the types of AI-driven technologies and digital tools currently adopted in healthcare contexts, and analyzes the outcomes they address at both the individual and organizational levels. It further explores the main barriers and facilitators affecting their implementation and adoption, paying specific attention to the degree of personalization and the quality of human–technology integration embedded in these interventions. Finally, it investigates how organizational dynamics and staff engagement strategies influence the effectiveness and sustainability of AI-based applications in promoting health and performance in the workplace.

## 2 Materials and methods

This scoping review was conducted following the methodological framework proposed by Arksey and O'Malley (27), further refined by Levac et al. (28) and the Joanna Briggs Institute (JBI) (29). The reporting of findings adheres to the PRISMA Extension for Scoping Reviews (PRISMA-ScR) guidelines (30).

This framework involves five key stages: identifying the research questions, identifying relevant studies, selecting studies based on inclusion criteria, charting the data, and finally collating, summarizing, and reporting the results. The refinements by Levac et al. (28) and the JBI (29) emphasize transparency, iterative team-based screening, and structured data synthesis, features that guided each phase of our review process.

### 2.1 Objectives and research questions

The primary objective of this scoping review is to systematically map existing evidence on AI-based digital technologies used to support performance management within workplace health promotion programs targeting healthcare workers. Specifically, the review aims to identify:

- *RQ1* The types of AI-driven technologies and digital tools used;
- *RQ2* The organizational and individual-level outcomes measured;
- *RQ3* The barriers and facilitators to implementation and adoption;
- *RQ4* The degree of personalization and human–technology integration;
- *RQ5* The role of organizational dynamics and engagement strategies in influencing effectiveness.

### 2.2 Eligibility criteria

To ensure methodological clarity and rigor, eligibility criteria were formulated using the PCC (Population–Concept–Context) framework, as recommended by International Consensus (29, 31). Table 1 summarizes how the three components of the PCC framework were operationalized in this study. This approach was selected because PCC is the most appropriate framework for scoping reviews aimed at mapping broad and heterogeneous bodies of evidence.

**Table 1:** Eligibility criteria based on the PCC framework.

**Element**	**Description**	**Inclusion criteria**	**Exclusion criteria**
Population	Healthcare workers involved in or targeted by digital health promotion interventions	Clinical and non-clinical staff working in healthcare settings (e.g., nurses, physicians, allied health professionals, administrative staff)	Non-healthcare workers; studies on general population, students, or patients
Concept	AI-based digital technologies used to support both performance management and workplace health promotion among healthcare workers	Studies examining AI tools such as machine learning algorithms, natural language processing (NLP), deep learning models, recommender systems, or predictive analytics used to support engagement, wellbeing, or performance	Studies not involving AI or lacking a workplace health promotion/performance component
Context	Workplace-based health promotion and performance monitoring programs in healthcare settings	Studies conducted in formal healthcare organizations (e.g., hospitals, clinics, health systems)	Non-workplace or informal care settings; interventions outside occupational contexts

The “Population” was defined as healthcare workers, including both clinical and non-clinical staff, targeted by AI-enabled health promotion interventions. The “Concept” focused on AI-based digital technologies designed to support both workplace health promotion and performance management. The “Context” was limited to formal healthcare settings where these technologies were applied.

Its structure allows for flexibility in defining complex concepts, such as AI-based workplace health promotion, and ensures transparent alignment between the research question, the screening strategy, and data extraction. In our review, the PCC framework supported the systematic identification of literature across health informatics, occupational health, organizational behavior, and digital health implementation.

This framework ensured consistency in study selection and supported the review's aim of capturing multidisciplinary perspectives while maintaining a clear focus on the healthcare workforce.

### 2.3 Search strategy

A comprehensive and systematic search strategy was developed to identify peer-reviewed and gray literature across fields such as occupational health, digital medicine, organizational psychology, health informatics, and human resource analytics.

Electronic searches were conducted in the following databases: PubMed; Scopus; Web of Science; PsycINFO; IEEE Xplore; Google Scholar (for gray literature and reports).

The search covered publications from January 2010 to July 2025, to reflect the rise in AI applications in occupational and digital health contexts. Only studies published in English were included.

Search terms combined controlled vocabulary (e.g., MeSH) and free-text keywords, using Boolean operators (AND, OR) and truncation. Key terms included: *artificial intelligence, workplace health promotion, healthcare workers, performance management, engagement, predictive analytics, wearable technology, chatbots, digital interventions, AI personalization*.

A full list of search strings per database is available in Supplementary Table S1.

### 2.4 Study selection

All references were imported into Rayyan software (32), and duplicates were removed. Study selection was conducted in two phases:

- Title and Abstract Screening: Two independent reviewers screened titles and abstracts for relevance based on the PCC framework. Studies not meeting inclusion criteria were excluded.
- Full-Text Review: Full-text articles were retrieved and assessed for inclusion. Discrepancies were resolved by consensus or by consulting a third reviewer.

A PRISMA-ScR flow diagram documents the screening and selection process, including the number of records identified, excluded, and included, along with reasons for full-text exclusions.

The final selection included studies that directly addressed the use, effectiveness, barriers, or enablers of AI-based digital technologies in workplace health promotion and performance optimization for healthcare workers.

## 3 Results

**Figure 1:**
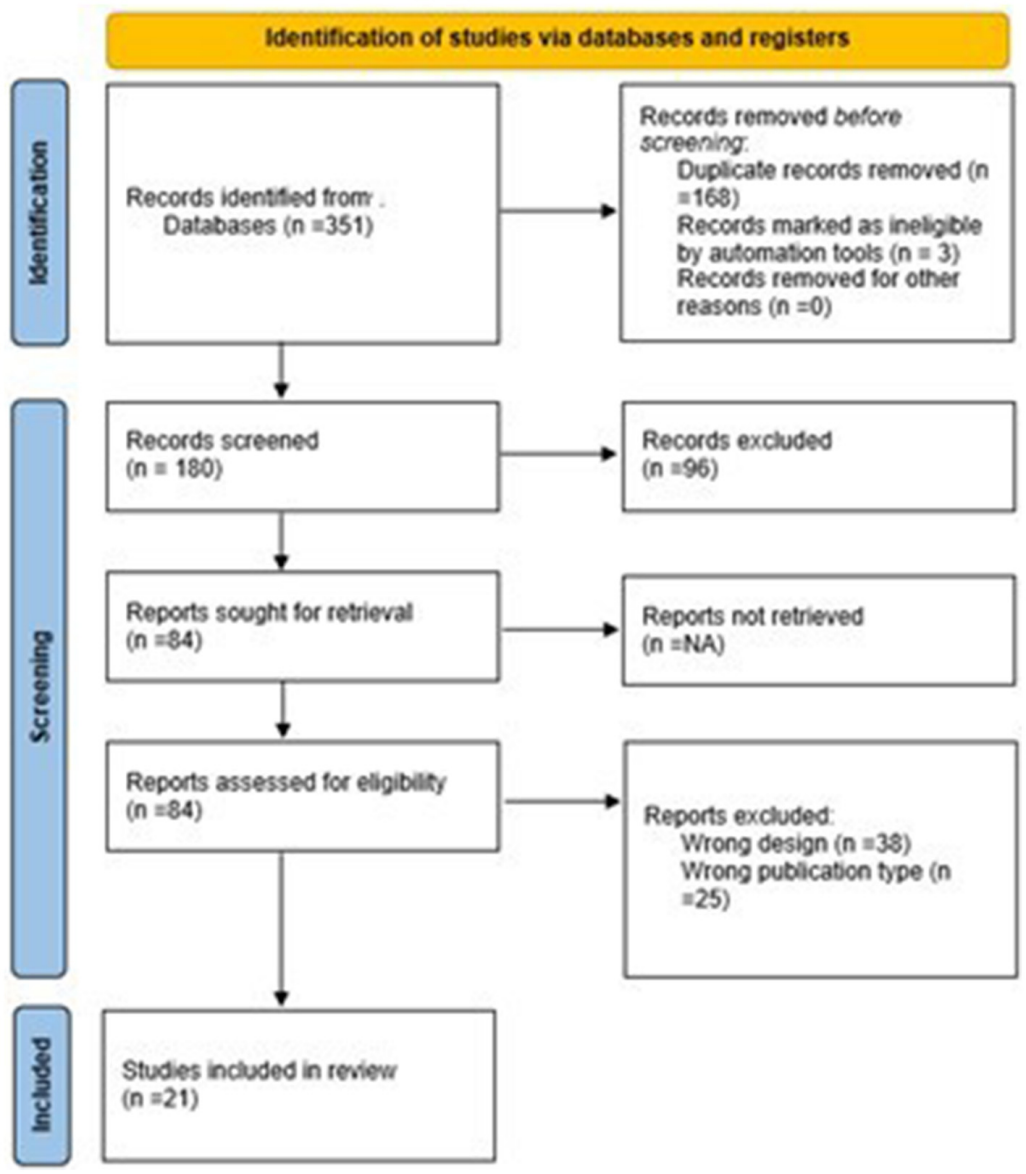
PRISMA-ScR flow diagram illustrating the identification, screening, eligibility, and inclusion of studies on AI-based digital technologies for performance management in workplace health promotion among healthcare workers.

A comprehensive search across several electronic databases initially yielded 351 records. The PRISMA flowchart (Figure 1) details the process of selecting relevant publications at each step of the review. After eliminating 168 duplicate entries and other 3 ineligible paper (editorials or commentaries), 180 records advanced to the screening phase. During this stage, 96 records were excluded based on their failure to meet the established inclusion criteria. This left 84 articles to be further assessed for eligibility.

In the final assessment phase, 63 full-text articles were excluded because they did not meet key eligibility requirements, specifically due to:

- lack of original data (e.g., reviews, opinion papers, conceptual frameworks),
- absence of exposure or outcome of interest (e.g., studies not analyzing AI-based documentation systems or not reporting relevant clinical/organizational outcomes),
- insufficient methodological detail (e.g., studies where the design, sample characteristics, or analytic methods were unclear or not extractable),
- irrelevance to the review scope (e.g., articles focusing solely on technical algorithm development without assessing real-world implementation or clinical documentation impacts).

A final total of 21 studies met all inclusion criteria and were incorporated into the qualitative and quantitative synthesis. All included studies were published in peer-reviewed academic journals. No gray literature sources (e.g., institutional reports, white papers, or non-peer-reviewed publications) met the inclusion criteria or were retained in the final selection.

### 3.1 Characteristics of included studies

The 23 studies included in this scoping review, published between 2015 and July 2025, demonstrate a heterogeneous range of research designs, AI technologies, and health promotion applications within healthcare workplaces (Table 2).

**Table 2:** Summary of study characteristics: design, AI technology type, health promotion focus, and target outcomes.

**Study**	**Study design**	**AI technology type**	**Health promotion focus**	**Target outcomes**
Adamopoulos et al. (33)	Quantitative, predictive analytics	Machine learning models (XGBoost, random forest, long short-term memory, natural language processing)	Hazard/burnout prediction, safety	Hazard prediction, burnout risk, incident reduction
Adile et al. (34)	Mixed-methods	Deep learning (deep learning-based adaptive safety monitoring)	Safety training, workplace health	Efficiency, accuracy, predictability
Agarwal et al. (35)	Randomized controlled trial	Automated text messaging, wellbeing platform	Mental health, engagement	Depression, anxiety, wellbeing
Anan et al. (36)	Randomized controlled trial	Chatbot	Musculoskeletal health	Pain reduction, adherence
Arboh et al. (37)	Quantitative (survey)	General AI integration	AI awareness, wellbeing	Informal learning, wellbeing
Bangani et al. (38)	Case study (quantitative)	Machine learning/artificial intelligence stress detection	Stress monitoring, burnout prevention	Stress prediction, burnout prevention
Bienefeld et al. (39)	Qualitative case study	AI for work design	Work conditions, stress reduction	Autonomy, skill diversity, flexibility, motivation, performance
Chang et al. (40)	Quantitative (pilot)	AI-led mental health app	Mental wellbeing (sleep, anxiety)	User engagement, anxiety, depression
Fragouli (41)	Mixed-methods case study	Chatbots, sentiment analysis	Digital empathy, wellbeing	Mental health, workload, emotional recognition
Khavandi et al. (42)	Multiple case study, mixed-methods	Autonomous clinical conversational assistant	Automation of routine tasks, staff wellbeing	Professional identity, wellbeing, work practices
Khavandi et al. (43)	Mixed-methods, multicentre	Autonomous clinical voice assistant	Automation, workflow change	Professional identity, wellbeing, workflow
Kumar et al. (44)	Quantitative (survey)	AI-driven operations management system	Operational efficiency, staff attitudes	Efficiency, staff attitudes
Kwong and Stafford (45)	Descriptive study	AI-based documentation	Administrative burden, documentation	Documentation metrics, empowerment
Lintz and Lintz (46)	Quantitative	AI-based hand hygiene monitoring	Infection prevention, workflow	Attitudes, satisfaction, healthcare-associated infection reduction
Namatovu and Kyambade (47)	Quantitative (survey)	General AI integration	Employee performance, skills	Performance, perceived ease of use, skills enhancement
Romero-Brufau et al. (48)	Mixed-methods, pre-post	AI-based clinical decision support	Care coordination, team dialog	Staff attitudes, care coordination
Rouhani et al. (49)	Mixed-methods	Deep learning (convolutional neural network) for emotion recognition	Human resource management, staff performance, emotion recognition	Model accuracy, staff performance, satisfaction
Turchioe et al. (50)	Quantitative (survey)	General AI + Digital Health (telehealth, portals, devices)	Burnout reduction, digital health support	Burnout, task support, staff retention
Yeh et al. (51)	Prospective cohort	Internet of Things-based temperature monitoring	Infection prevention, early warning	Measurement completion, early detection
Yntig (52)	Quantitative, data mining	Data mining/artificial intelligence	Work performance	Performance, motivational factors
Zheng et al. (53)	Quantitative cross-sectional	General AI applications in clinical settings	Workforce sustainability & performance enhancement	Job performance

The majority employed quantitative methodologies, including survey-based studies (47, 50, 53), pilot evaluations (40), and predictive modeling (33), while a smaller number adopted randomized controlled trials (35, 36), qualitative case studies (39), or mixed-methods approaches (34, 41, 43). The target populations across studies included nurses, physicians, residents, informaticists, and healthcare administrators, reflecting the breadth of roles potentially impacted by AI-enabled tools in clinical and organizational environments.

Artificial intelligence technologies examined in the included studies ranged from general or unspecified AI applications (37, 53) to more clearly defined systems such as machine learning algorithms, natural language processing, and deep learning models (33, 49). Specific implementations included autonomous voice assistants (43), clinical chatbots (36, 41), AI-based decision support systems (48), predictive analytics for burnout and safety (38), and AI-driven operations platforms (44). Some studies investigated AI within hybrid digital ecosystems, such as telehealth services (50), automated documentation systems (45), and Internet of Things-enabled monitoring tools (51).

In terms of health promotion focus, the literature emphasized three primary dimensions: enhancing occupational health and safety, supporting mental wellbeing and emotional resilience, and improving organizational efficiency. Several studies framed AI as a supportive tool for reducing healthcare worker burnout, managing stress, and promoting informal learning and engagement (35, 37, 41). Others positioned AI as an enabler of workflow optimization and professional identity reinforcement (42, 44), while some addressed public health and preventive goals, such as infection control (46, 51) and ergonomics-related outcomes (34, 36).

The outcomes measured were equally diverse. Across studies, job performance emerged as a recurrent metric, often operationalized through composite indices (53), staff self-assessments (47), or patient-centered indicators such as satisfaction and follow-up (50). Mental health outcomes—including stress, anxiety, and depressive symptoms—were also commonly assessed (35, 40). Other relevant metrics included staff engagement (41), task-specific performance (43), documentation burden (45), and infection-related indicators (46). Several studies introduced mediating variables such as perceived ease of use (47), informal learning behavior (37), or patient support for AI usage (53), which were integrated into structural models to explore indirect effects and contextual moderators.

During the data extraction process, each study was reviewed for its methodological design, AI technology type, health promotion aims, and reported outcomes. Variables were charted into a comparative matrix to facilitate thematic synthesis, with particular attention to the intersection between technological implementation and workplace dynamics. This allowed for identification of patterns across different healthcare contexts, including how AI interventions were integrated into clinical routines, how their impact was mediated by organizational culture, and how specific AI functions, such as automation, recommendation, or predictive modeling, were aligned with performance, wellbeing, or safety goals.

Overall, the body of evidence reflects a growing, though methodologically uneven, field of research exploring AI's role in workforce health promotion. While the majority of studies report positive or promising effects, the heterogeneity in study designs, technological maturity, and measurement tools calls for cautious interpretation. Moreover, despite the rapid acceleration of AI integration in healthcare, relatively few studies engage critically with long-term outcomes, ethical implications, or the sustainability of AI-supported health promotion programs in real-world settings.

### 3.2 Effects on worker health and organizational outcomes

The included studies reported a wide range of effects linked to the implementation of AI-based technologies in healthcare, with impacts observed both at the individual level (i.e., worker health and wellbeing) and the organizational level (i.e., efficiency, workflow, and productivity). While clinical and psychological indicators such as stress, burnout, and mental health were frequently examined, several studies also assessed operational outcomes, highlighting how AI influences work environments, resource allocation, and administrative performance (Table 3).

**Table 3:** Reported effects of AI-based technologies on worker health and organizational outcomes.

**Study**	**AI technology**	**Worker health outcomes**	**Organizational metrics**	**Engagement/adherence**
Adamopoulos et al. (33)	ML for burnout/hazard prediction	28% burnout and 35% incident reduction	Not reported	Not reported
Agarwal et al. (35)	Digital engagement (RCT)	Modest improvements in depression and anxiety	Not reported	Not reported
Anan et al. (36)	Chatbot (RCT)	Pain reduction (OR = 6.36)	Not reported	92% adherence
Bangani et al. (38)	Wearable stress detection	Stress and burnout prevention	Not reported	Not reported
Chang et al. (40)	AI mental health app	Reduced anxiety and depression	Not reported	80.1% used ≥2 sessions
Fragouli (41)	Digital empathy tools	Early stress detection, mixed reception	Not reported	Not reported
Khavandi et al. (42, 43)	Voice assistant	Wellbeing and workflow support	60% reduction in clinician consults	Not reported
Kumar et al. (44)	AI operations management (survey)	Improved staff attitudes	Increased efficiency	Not reported
Kwong and Stafford (45)	AI documentation	Not reported	Improved documentation metrics	Not reported
Namatovu and Kyambade (47)	General AI integration (survey)	Performance and skills enhancement	Not reported	Not reported
Rouhani et al. (49)	Emotion recognition (CNN)	Enhanced performance and satisfaction	92% model accuracy	Not reported
Turchioe et al. (50)	Digital health/AI tools	Burnout reduction	Increased task support; 38% AI adoption	Not reported
Yeh et al. (51)	IoT temperature monitoring	Early fever detection	Increased measurement completion	85% completion rate

CNN, Convolutional Neural Network; ML, Machine Learning; IoT, Internet of Things; RCT, Randomized Controlled Trial.

At the individual level, multiple interventions aimed to improve mental health, emotional resilience, and stress management. For instance, Chang et al. (40) found that an AI-supported mental health app significantly reduced anxiety and depression, with a high engagement rate (80.1%). Similarly, Agarwal et al. (35) reported modest improvements in depression and anxiety through a randomized controlled trial on digital engagement, though adherence data was not disclosed. Studies using predictive or monitoring tools also showed favorable outcomes: Adamopoulos et al. (33) reported a 28% reduction in burnout and a 35% decrease in clinical incidents through machine learning-driven hazard prediction, while Bangani et al. (38) described stress detection via wearable sensors as effective for burnout prevention.

Pain reduction and physiological monitoring also featured prominently. Anan et al. (36) demonstrated that a chatbot-based intervention achieved high adherence (92%) and a significant reduction in musculoskeletal pain (OR = 6.36). Yeh et al. (51), focusing on early warning systems, showed that IoT-based temperature monitoring led to improved fever detection and an 85% measurement completion rate. Studies by Rouhani et al. (49) and Fragouli (41) explored emotional and relational dimensions, using AI for emotion recognition and digital empathy, respectively. The former reported enhanced performance and satisfaction, while the latter found early stress detection but ambivalent user acceptance.

Organizational outcomes were reported in a growing subset of studies that examined workflow optimization, administrative efficiency, and operational metrics. Khavandi et al. (42, 43) found that the use of an autonomous voice assistant led to a 60% reduction in clinician-led consults, improving workflow and reducing staff burden. Similarly, Kumar et al. (44) reported perceived improvements in staff attitudes and efficiency following the implementation of an AI-based operations management system.

Several studies also reported improvements in documentation and resource management. For example, Kwong and Stafford (45) observed enhanced documentation metrics following the implementation of AI-based transcription tools. Other studies extended these findings by examining skills development, recruitment, and digital readiness: Namatovu and Kyambade (47) showed that AI adoption was associated with improved performance and enhanced professional skills among healthcare workers.

Together, these findings indicate that AI technologies hold promise not only in promoting individual health and psychological resilience but also in transforming workplace operations and institutional effectiveness. Nonetheless, variability in reporting standards, lack of long-term outcome tracking, and underreporting of user engagement and ethical concerns point to the need for more rigorous, integrative studies that assess both personal and organizational dimensions of AI adoption in healthcare settings.

### 3.3 Thematic analysis: AI-enabled health monitoring and intervention

One of the dominant thematic clusters emerging from the included literature pertains to AI-enabled health monitoring and intervention systems, particularly those designed for stress and burnout prevention in healthcare workers. These systems integrate wearable technologies, predictive analytics, biometric data, and emotion recognition to provide early detection of psychological strain and facilitate targeted interventions. The studies grouped under this theme varied in terms of technological complexity, delivery formats, and contextual applications, but they all converged on the goal of enhancing wellbeing and resilience among healthcare professionals (Table 4).

**Table 4:** Integrated overview of AI applications in worker health monitoring and performance optimization.

**Study**	**AI technology**	**Implementation approach**	**Success factor**	**Challenges**
Adamopoulos et al. (33)	Machine learning models, NLP	Predictive analytics, real-time data	High accuracy, incident and burnout reduction	Not reported
Agarwal et al. (35)	Automated text messaging	RCT, wellbeing platform	Modest improvement in depression and anxiety	Smartphone access required
Anan et al. (36)	Chatbot	Standalone app, RCT	Significant pain reduction, high adherence	Minimal human support
Bangani et al. (38)	AI stress detection	Wearables, case study	Effective stress prediction, burnout prevention	Not reported
Chang et al. (40)	AI-led mental health app	Standalone app, pilot	High engagement, repeat use	Limited to moderate anxiety, pandemic context
Fragouli (41)	Chatbots, sentiment analysis	Digital wellness tools	Early stress detection, personalized nudges	Employee ambivalence, privacy concerns
Khavandi et al. (42, 43)	Autonomous voice assistant	NHS integration, multicentre	Reduces administrative burden, supports managers	Frontline resistance, workflow fit
Kumar et al. (44)	AI-driven operations system	Survey, cross-sectional	Improved efficiency, positive staff attitudes	Need to address perceptions
Kwong and Stafford (45)	AI-based documentation	Not mentioned	Administrative burden reduction	Not reported
Namatovu and Kyambade (47)	Not mentioned	Survey	Performance improvement, skills enhancement	Need for training, usability
Rouhani et al. (49)	Deep learning (CNN)	Cameras, emotion recognition	High accuracy, enhanced staff performance and satisfaction	Reliance on facial recognition
Turchioe et al. (50)	Not mentioned	Survey	Task support, burnout reduction	Low AI usage
Yeh et al. (51)	IoT-based temperature monitoring	Wearables, cohort	Increased measurement completion	Not reported

Several interventions adopted machine learning and predictive analytics to proactively address stress-related risks. Adamopoulos et al. (33) employed a suite of machine learning models—including natural language processing and real-time data collection—to predict burnout and hazardous events with high accuracy, resulting in measurable reductions in clinical incidents. Similarly, Bangani et al. (38) used wearable-based stress detection to enable early intervention in high-risk staff groups, reporting effective prediction outcomes, although details on implementation and engagement were limited.

Other studies explored mobile and standalone digital health applications aimed at improving mental health outcomes. Anan et al. (36), for instance, developed a chatbot-based application to manage musculoskeletal pain, achieving statistically significant reductions in symptoms and high adherence, with minimal human involvement.

Chang et al. (40) introduced an AI-led mental health app focused on moderate anxiety symptoms during the COVID-19 pandemic, which showed high levels of repeated use and engagement. In a similar domain, Agarwal et al. (35) utilized automated text messaging via a wellbeing platform in a randomized controlled trial, which resulted in modest improvements in depression and anxiety. However, the intervention required consistent smartphone access, raising questions about digital equity.

Emotion recognition emerged as another promising but contested application area. Rouhani et al. (49) implemented a deep learning model using facial recognition via camera systems to assess emotional states in real time, achieving high accuracy and reported improvements in staff performance. Fragouli (41) combined sentiment analysis and chatbots to deliver personalized nudges for stress management, but also highlighted user ambivalence and concerns about surveillance and emotional intrusion.

Complementing these digital psychological interventions, Yeh et al. (51) investigated a more physiological dimension of health monitoring using Internet of Things (IoT)-enabled temperature sensing devices. While not directly targeting mental health, the system supported early detection of febrile symptoms in clinical staff, contributing to occupational safety and continuity of care through increased adherence to measurement protocols.

Taken together, these studies reflect the growing reliance on AI-enabled systems to mitigate stress, monitor wellbeing, and facilitate early intervention among healthcare workers. While the majority of tools show encouraging outcomes, particularly in terms of engagement and predictive capacity, their success appears to hinge on factors such as ease of use, ethical design, personalization, and the level of required human oversight. Concerns related to data privacy, emotional surveillance, and technological overreach remain salient, underscoring the importance of transparent, participatory approaches in the development and implementation of AI-supported mental health tools in healthcare environments.

In addition to mental health and wellbeing interventions, a second thematic cluster centers on AI-based performance optimization tools aimed at improving organizational efficiency, reducing administrative burden, and enhancing staff capabilities. These tools, while diverse in design and application, share a common objective: to support healthcare professionals in delivering more effective care by automating tasks, optimizing workflow, and facilitating strategic decision-making.

Multiple studies explored AI integration into administrative and operational systems. Khavandi et al. (42, 43) examined autonomous voice assistants deployed across multiple NHS centers, reporting a substantial reduction in clinician-led consultations and improved managerial oversight. However, they also identified frontline resistance and workflow integration challenges as notable barriers to successful adoption. Similarly, Kumar et al. (44), through a cross-sectional survey, highlighted how AI-driven operations management systems led to measurable gains in staff efficiency and positive shifts in attitudes, though underscored the need for cultural and perceptual adaptation among users.

Administrative automation was also a recurring theme. Kwong and Stafford (45) evaluated an AI-based documentation system aimed at reducing the administrative workload, noting positive documentation outcomes, although user feedback and long-term impact were not systematically reported. Turchioe et al. (50) confirmed similar findings, indicating that digital health tools supported task execution and mitigated burnout, albeit within the context of relatively low AI uptake (38%).

Some studies also positioned AI as a strategic enabler of leadership and resource management. However, they emphasized persistent concerns around ethical implementation and professional resistance.

Digital platforms and behavioral tracking were also leveraged to optimize engagement. More recently, Namatovu and Kyambade (47) examined general AI usage in Ugandan hospitals and reported enhancements in staff performance and skill acquisition, though the need for ongoing training and usability concerns were emphasized.

Collectively, these studies illustrate the multifaceted role of AI in enhancing organizational performance. While evidence points to measurable improvements in efficiency, documentation, leadership, and administrative functioning, the success of these technologies is contingent on several factors: alignment with clinical workflows, stakeholder engagement, data ethics, and technical readiness. The variation in implementation depth and user perceptions across settings highlights the importance of context-specific strategies for AI deployment in healthcare environments.

### 3.4 Summary of findings by research question

This scoping review set out to explore the intersection of AI-based digital technologies with workplace health promotion and performance management among healthcare workers. The analysis was guided by five research questions (RQ1–RQ5), each addressing a different dimension of how AI tools are designed, implemented, and evaluated in this context. Below is a synthesis of key findings structured around each research question, highlighting the diversity of technologies used, the range of measured outcomes, as well as critical insights into adoption dynamics and human–AI integration in healthcare settings.

#### 3.4.1 RQ1—The types of AI-driven technologies and digital tools used

The reviewed studies identified a broad array of AI-driven technologies applied in healthcare workplace settings. These included machine learning models and predictive analytics for risk detection and hazard prevention, natural language processing and deep learning for emotion recognition, chatbots for self-monitoring and mental health support, AI-based documentation and workflow optimization tools, and autonomous voice assistants. Some interventions were integrated into broader ecosystems such as mobile health platforms, Internet of Things (IoT) devices, and telehealth systems. The technological diversity reflects the multidimensional applications of AI to address both performance and wellbeing among healthcare workers.

#### 3.4.2 RQ2—The organizational and individual-level outcomes measured

Outcomes reported across the studies spanned both individual and organizational domains. At the individual level, common outcomes included improvements in mental health (e.g., reductions in anxiety, stress, depression), physical health (e.g., pain reduction), and staff satisfaction or perceived wellbeing. At the organizational level, several studies assessed improvements in workflow efficiency, documentation accuracy, task automation, and clinician workload. Engagement and adherence metrics—such as app usage rates and completion of intervention activities—were also reported, though inconsistently. Together, the outcomes demonstrate a dual focus on enhancing employee health and supporting operational effectiveness.

#### 3.4.3 RQ3—The barriers and facilitators to implementation and adoption

Thematic analysis revealed several barriers to adoption, including concerns over data privacy, emotional surveillance, low digital literacy, and misalignment with clinical workflows. Resistance from frontline staff and a lack of algorithm transparency were also noted. Conversely, key facilitators included the perceived usefulness of AI tools, improved task efficiency, personalization of interventions, and strong institutional support. Studies also highlighted that successful implementation was often dependent on prior engagement with end users, adequate training, and integration with existing digital infrastructures.

#### 3.4.4 RQ4—The degree of personalization and human–technology integration

Several interventions offered varying degrees of personalization, such as adaptive content delivery, individualized feedback, and emotion-aware interfaces. Chatbots and wellbeing apps were often designed to respond dynamically to user input or physiological data. However, explicit evaluation of human–technology integration was limited. While some studies implemented co-adaptive systems or user-responsive interfaces, few addressed user experience systematically. This suggests that personalization is an emerging strength of AI-based tools, though the degree of integration with human workflows remains underexplored.

#### 3.4.5 RQ5—The role of organizational dynamics and engagement strategies in influencing effectiveness

Organizational context was a major determinant of intervention success. Studies showed that leadership support, clear governance, and alignment with existing workflows were central to effective adoption. In contrast, poorly defined AI roles, lack of staff involvement, or cultural resistance reduced impact. Engagement strategies such as participatory co-design, transparent communication, and training improved trust and uptake. The review underscores the need to frame AI implementation within broader organizational change processes, where human and structural dynamics actively shape technological outcomes.

## 4 Discussion

This scoping review mapped the state of the art regarding artificial intelligence (AI)–based digital technologies for workplace health promotion and performance management among healthcare workers. Two broad domains of application emerged: (i) AI-enabled health monitoring and interventions, primarily aimed at preventing stress, burnout, and mental health decline, and (ii) AI-driven performance optimization, focusing on workflow efficiency, administrative support, and organizational sustainability. While the findings reveal promising applications, they also highlight substantial methodological, ethical, and practical challenges that require critical reflection.

Evidence consistently shows that AI supports healthcare worker wellbeing by enabling early detection of stress, anxiety, and physical health problems, and by providing personalized interventions. Tools such as chatbots, emotion recognition algorithms, and mobile health applications have demonstrated benefits in reducing anxiety, depression, pain, and improving adherence to self-care routines (36, 40). Wearable-based stress detection and Internet of Things (IoT) systems further enhance real-time health monitoring (38, 51). Predictive models leveraging natural language processing and machine learning reduce burnout incidence and workplace hazards by identifying at-risk staff (33).

At the same time, the literature highlights persistent concerns. Emotion recognition and biometric monitoring raise questions of privacy, autonomy, and potential surveillance (49, 54). Ambivalence among employees, especially in relation to sentiment analysis and digital empathy tools (41), indicates that perceived intrusiveness may hinder adoption. Moreover, most studies are short-term, often pilots, and rarely address the long-term sustainability of benefits.

AI-enabled stress and burnout monitoring has shown encouraging results in reducing stress and enhancing professional fulfillment (55, 56). Wearable devices and machine learning algorithms detect stress patterns in real time (38), while mindfulness-based resilience training provides broader benefits compared to digital-only tools (57). Organizational interventions, such as schedule adjustments, are also effective in reducing stress (58), and sustained reductions in burnout and perceived stress have been observed when organizational support is included (59). Still, data privacy and ethical implementation remain major barriers (5). Recent research addresses these challenges through privacy-preserving AI approaches, such as federated learning and differential privacy, which improve security without compromising predictive accuracy (60–62). Responsible AI frameworks emphasize transparency, accountability, and mitigation of algorithmic bias (63–66). These approaches highlight the need to balance predictive accuracy with ethical safeguards.

The second major domain of evidence concerns organizational performance. Automated documentation, operations management systems, and voice assistants have reduced administrative burden, improved workflow efficiency, and supported decision-making (42–45). Reviews and survey studies confirm gains in productivity, skills enhancement, and ease of use when AI is integrated into work systems (47, 49). At the organizational level, AI adoption improves resource allocation, task distribution, and reduces overtime (49, 67).

However, these benefits are not uniform. Barriers include frontline resistance, poor alignment with workflows, and inequitable access to digital infrastructure (43). Engagement is variable, with some studies reporting low uptake despite demonstrated efficiency gains (50).

The literature emphasizes the role of leadership, organizational culture, and digital readiness as key enablers of AI adoption. Change leadership fosters engagement and performance (68), while cultures that support data-driven decision-making encourage innovation and collaboration (69, 70). Conversely, perceptions of job insecurity or lack of usefulness hinder adoption (71). Effective integration therefore requires transparent communication, continuous training, and supportive organizational environments (72, 73).

Healthcare workers' perceptions significantly shape AI adoption and its outcomes. Studies show that performance expectancy, effort expectancy, and social influence increase adoption intentions (74, 75). Knowledge exposure and AI-focused training correlate with more positive attitudes (76, 77). Nevertheless, professionals still express concerns about privacy, accountability, and safety (78, 79). To strengthen adoption, healthcare institutions should prioritize functionality, usability, and workforce training (80, 81).

Methodologically, most studies are observational or survey-based, with few randomized controlled trials (35, 36). Outcome measures are highly heterogeneous, spanning mental health, job satisfaction, workflow efficiency, patient satisfaction, and technical accuracy. This lack of standardization hampers comparability and generalization. Moreover, long-term outcomes are rarely assessed, raising concerns about sustainability.

Workplace health promotion research shows that AI has potential for individualized risk prediction and targeted interventions (19). Yet, standardized metrics are needed to improve comparability (20). Traditional WHP programs improve vitality, performance, and reduce absenteeism (82), but digital health interventions often face engagement challenges (83). Evidence on ROI remains inconsistent, as intangible benefits are harder to monetize (84, 85).

Recent evaluations suggest pragmatic trial designs are well-suited for assessing AI in real-world healthcare (86, 87). While RCTs show promising outcomes (88), complementary pragmatic and embedded trials are needed for external validity (89). Value Sensitive Design and human-centered playbooks are increasingly proposed to ensure alignment with workflow and values (90–92).

Finally, human–technology interaction emerges as a critical determinant. Technical accuracy alone does not ensure adoption; rather, personalization, transparency, and co-design improve usability and trust (93). Stress monitoring and wellness tools must adapt to diverse worker needs, or risk abandonment. Training and digital literacy programs are crucial to build confidence (94–96). Research stresses balancing automation with human oversight to preserve accountability and trust (97, 98). Human-centered AI frameworks and explainability models show how autonomy can be safeguarded while optimizing performance (99–103). Co-design and training remain decisive facilitators of adoption (104–107).

Future research should prioritize methodological rigor and long-term evaluation of AI-based interventions in workplace health promotion and performance management. Most of the current evidence is short-term and heterogeneous, limiting generalizability. Pragmatic trials, hybrid designs, and embedded implementation studies are needed to assess real-world impact, sustainability, and cost-effectiveness (86, 88).

A key priority is the development of standardized frameworks for outcome measurement that integrate individual health indicators, organizational performance metrics, and ethical dimensions of AI use. Without harmonization, the evidence base will remain fragmented and difficult to translate into practice. Multidimensional evaluation strategies, capturing both clinical and organizational outcomes, are essential to determine return on investment and broader system-level impacts.

Research must also advance privacy-preserving AI approaches, such as federated learning and edge computing, to address ongoing concerns about data protection, autonomy, and algorithmic bias (60, 61). Interdisciplinary studies combining computer science, occupational psychology, and organizational behavior are particularly well-suited to address these challenges.

Several interventions included in this review were implemented during the COVID-19 pandemic, a period marked by exceptional strain on healthcare systems and heightened psychological burden among health workers. While this context may have amplified engagement with digital tools or influenced outcomes such as burnout and stress, it also reflects a real-world testing ground for AI-enabled interventions under acute conditions. However, this introduces a potential limitation in terms of external validity. Where possible, studies were labeled according to their temporal context (pre-, peri-, or post-COVID), and findings were interpreted with attention to their pandemic setting. Future research should further explore whether observed benefits persist in non-pandemic scenarios and more stable work environments.

Furthermore, future investigations should examine the sociotechnical dynamics of AI adoption. This includes understanding how leadership, organizational culture, and employee attitudes interact with AI-driven changes. Comparative studies across countries and healthcare systems would shed light on contextual moderators, particularly in low- and middle-income countries where digital infrastructure may lag.

Ethical aspects such as data governance, user consent, and algorithmic fairness were only sporadically reported across included studies. While these were not the focus of this scoping review, future research should systematically assess such dimensions to ensure responsible and trustworthy AI adoption in occupational health contexts.

Finally, participatory design and human-centered AI remain critical directions. Co-design approaches involving healthcare workers in the development and adaptation of AI systems could improve usability, trust, and long-term adoption. Research should evaluate strategies that enhance digital literacy, workforce training, and organizational readiness, ensuring that technological innovation translates into tangible improvements in healthcare worker wellbeing and organizational sustainability.

## 5 Conclusions

This scoping review highlights both the potential and the challenges of integrating AI-based digital technologies into workplace health promotion and performance management for healthcare workers. Across diverse applications, from stress detection and mental health monitoring to workflow optimization and operations management, AI demonstrates measurable benefits for individual wellbeing, organizational efficiency, and professional sustainability.

At the same time, ethical concerns, methodological limitations, and variability in adoption underscore the need for cautious, evidence-based implementation. Technical accuracy alone is insufficient; personalization, transparency, and human-centered design are decisive for success. Strong leadership, organizational support, and continuous workforce training further determine whether AI becomes a sustainable tool for improving healthcare work environments.

Overall, AI holds considerable promise for transforming workplace health promotion in healthcare. However, its effectiveness will depend on aligning technological innovation with ethical safeguards, robust evaluation frameworks, and the lived realities of healthcare workers. By embedding AI within supportive organizational cultures and prioritizing worker wellbeing alongside performance outcomes, healthcare systems can harness AI to achieve both human and organizational flourishing.

Future research should also compare AI-based approaches with traditional models of health care management and performance management to evaluate whether and how AI provides additional value.
