# Supplementary material for: Personalized AI for workplace health promotion: performance management and healthcare worker engagement through digital analytics
Source: Front Public Health. 2026 Jan 13;13:1718474. doi: 10.3389/fpubh.2025.1718474 (PMC12835227; doi:10.3389/fpubh.2025.1718474)
Supplement: Supplementary file 1 [file Table_1.docx]

**Table S1. Search Strategy Across Databases**

| **Database** | **Search String** | **Filters** |
| --- | --- | --- |
| **PubMed** | ("artificial intelligence" OR "machine learning" OR "predictive analytics") AND ("workplace health promotion" OR "occupational health" OR "employee wellness") AND ("healthcare workers" OR "nurses" OR "physicians") | English, 2010–2025, Humans |
| **Scopus** | TITLE-ABS-KEY(("artificial intelligence" OR "machine learning" OR "chatbots") AND ("employee health" OR "workplace promotion") AND ("healthcare personnel" OR "hospital staff")) | English, 2010–2025, Article |
| **Web of Science** | TS=(("AI" OR "digital health" OR "wearable technology") AND ("performance management" OR "employee engagement") AND ("clinical staff" OR "healthcare workforce")) | English, 2010–2025, Articles, Papers |
| **PsycINFO** | (DE "Artificial Intelligence" OR DE "Digital Technologies") AND (DE "Workplace Health Promotion" OR DE "Occupational Wellbeing") AND (DE "Healthcare Workers" OR DE "Medical Staff") | English, 2010–2025, Peer-reviewed Journals |
| **IEEE Xplore** | ("AI-based systems" OR "personalized AI") AND ("employee health tracking" OR "occupational health") AND ("healthcare professionals" OR "clinical environments") | English, 2010–2025, Healthcare domain only |
| **Google Scholar** | "AI for workplace health promotion" OR "digital analytics in healthcare" OR "employee performance AI healthcare" | First 200 results, relevance ranked |
